# National variation in the delivery of radiation oncology procedures in the non‐facility‐based setting

**DOI:** 10.1002/cam4.4028

**Published:** 2021-06-02

**Authors:** Luca F. Valle, Fang‐I Chu, Palak Kundu, Stephanie M. Yoon, Travis Gilchrist, Michael L. Steinberg, Ann C. Raldow

**Affiliations:** ^1^ Department of Radiation Oncology University of California, Los Angeles Los Angeles CA USA

**Keywords:** radiation therapy, behavioral science, clinical management, radiotherapy, registries

## Abstract

**Purpose:**

Though utilization of medical procedures has been shown to vary considerably across the United States, similar efforts to characterize variation in the delivery of radiation therapy (RT) procedures have not been forthcoming. Our aim was to characterize variation in the delivery of common RT procedures in the Medicare population. We hypothesized that delivery would vary significantly based on provider characteristics.

**Methods:**

The Centers for Medicare and Medicaid Services (CMS) Physician and Other Supplier Public Use File was linked to the CMS Physician Compare (PC) database by physician NPI to identify and sum all treatment delivery charges submitted by individual radiation oncologists in the non‐facility‐based (NFB) setting in 2016. Multivariable logistic regression analysis was carried out to determine provider characteristics (gender, practice rurality, practice region, and years since graduation) that predicted for the delivery of 3D conformal RT (3DCRT), intensity modulated RT (IMRT), stereotactic body RT (SBRT), stereotactic radiosurgery (SRS), low dose rate (LDR) brachytherapy, and high dose rate (HDR) brachytherapy delivery in the Medicare patient population. The overall significance of categorical variables in the multivariable logistic regression model was assessed by the likelihood ratio test (LRT).

**Results:**

In total, 1,802 physicians from the NFB practice setting were analyzed. Male gender predicted for greater LDR brachytherapy delivery (OR 8.19, 95% CI 2.58–26.05, *p* < 0.001), but not greater delivery of other technologies. Metropolitan practice was the only predictor for greater HDR brachytherapy utilization (OR 12.95, 95% CI 1.81–92.60, *p* = 0.01). Practice region was predictive of the delivery of 3DCRT, SRS and SBRT (*p *< 0.01, *p* < 0.001, and *p* < 0.001, respectively). With the Northeast as the reference region, 3DCRT was more likely to be delivered by providers in the South (OR 1.33, 95% CI 1.09–1.62, *p* < 0.01) and the West (OR 1.38, 95% CI 1.11–1.71, *p* < 0.01). At the same time, SRS use was less likely in the Midwest (OR 0.71, 95% CI 0.55–0.91, *p* < 0.01), South (OR 0.49, 95% CI 0.40–0.61, *p* < 0.001), and West (OR 0.43, 95% CI 0.34–0.55, *p* < 0.001). SBRT, on the other hand, was more commonly utilized in the Midwest (OR 2.63, 95% CI 1.13–6.13, *p* = 0.03), South (OR 3.44, 95% CI 1.58–7.49, *p* < 0.01), and West (OR 4.87, 95% CI 2.21–10.72, *p* < 0.001). HDR brachytherapy use was also more likely in the Midwest (OR 1.97, 95% CI 1.11–3.49, *p* = 0.02) and West (OR 1.87, 95% CI 1.08–3.24, *p* = 0.03). While the degree held by the billing physician did not predict for delivery of a given procedure, greater years since graduation was related to decreased likelihood of SBRT use (OR 0.98, 95% CI 0.96–0.99, *p* < 0.001) and increased likelihood of LDR brachytherapy use (OR 1.02, 95% CI 1.00–1.04, *p* = 0.02).

**Conclusions:**

Substantial geographic variation in the use of specific RT technologies was identified. The degree to which this variation reflects effective care, preference‐sensitive care, or supply‐sensitive care warrants further investigation.

## Graphical abstract

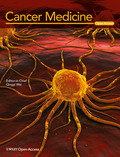

## 1 INTRODUCTION

Much attention has been focused on the significant variation in clinical practice that exists in the United States healthcare system, particularly with regard to highly‐reimbursed procedures.^1^, ^2^ The field of oncology has not been exempt from these perplexing patterns of care, and significant geographic variation has been reported in the management of very common malignancies, including prostate cancer.^3^

However, despite the fact that an estimated 50% of all cancer patients will undergo radiation therapy (RT) during the course of their disease,^4^ a broader analysis of variation across RT procedures has yet to be undertaken using robust national databases. Accordingly, predictors of RT procedures and technologies also remain poorly characterized. A more sophisticated understanding of how utilization of RT procedures varies according to the characteristics of the radiation oncology providers prescribing them would enable policymakers to focus on cost‐saving strategies in a field where technological innovation and improvements in oncologic outcomes are sometimes linked to high costs. Moreover, as the Radiation Oncology Alternative Payment Model (RO‐APM) begins to take shape,^5^ it becomes important to clarify utilization of highly reimbursed RT procedures across all disease.

In this study, we sought to better characterize the delivery of common RT procedures in the United States. We hypothesized that significant variation would exist in the delivery of RT procedures at non‐facility‐based freestanding centers. We examined the ways in which provider gender, practice rurality, practice region, and years since graduation predict for the use of specific RT procedures in the Medicare patient population.

## 2 METHODS

### 2.1 CMS databases

**FIGURE 1:**
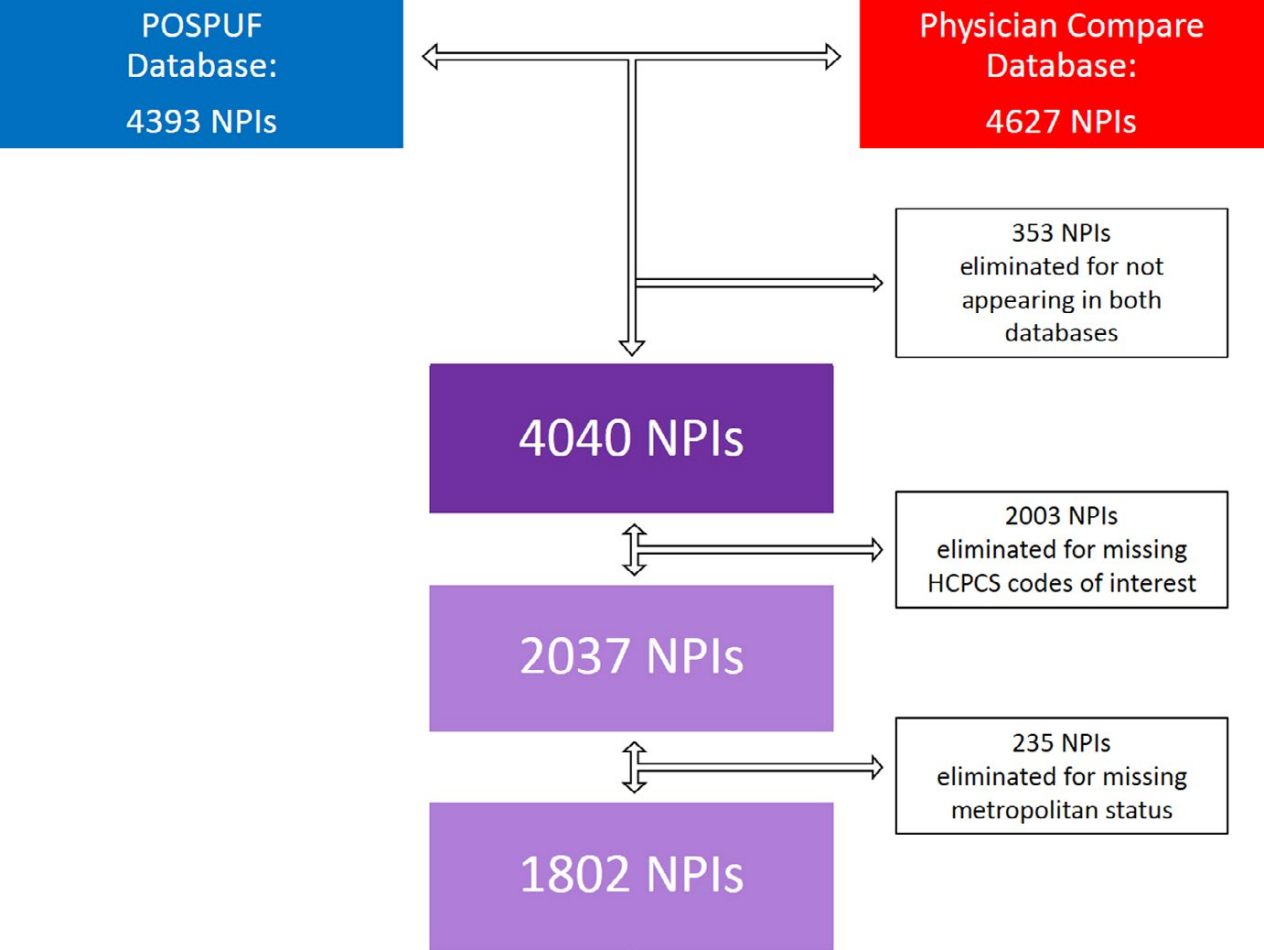
National provider identifiers (NPIs) linked and included for analysis. Flow chart depicts evaluable NPIs after linking and exclusion for missing data. Radiation Oncology NPIs identified in the POSPUF database are indicated in blue. Radiation Oncology NPIs in the Physician Compare database are indicated in red. 4040 NPIs were successfully linked. 1802 Unique NPIs were ultimately included in the analysis after eliminating NPIs on the basis of failing to appear in both databases, missing HCPCS codes of interest, and missing metropolitan status. POSPUF: Physician and Other Supplier Public Use File, NPI: National Provider Identifier, HCPCPS: Healthcare Common Procedure Coding System

We utilized the Centers for Medicare and Medicaid Services (CMS) Physician and Other Supplier Public Use File (POSPUF) database to identify charges submitted by individual radiation oncologists for the year 2016. First published in 2014, this database contains the Healthcare Common Procedure Coding System (HCPCS) codes submitted by each Medicare provider for reimbursement according to their unique National Provider Identifier (NPI).^6^ The POSPUF database was linked to the CMS Physician Compare (PC) database according to NPI in order to augment demographic information about the providers. Entries that could not be linked or lacked our prespecified variables of interest were excluded according to Figure 1.

From the POSPUF database, daily treatment delivery HCPCS codes for 3D‐conformal RT (3DCRT), intensity modulated RT (IMRT), stereotactic radiosurgery (SRS), stereotactic body RT (SBRT), low dose rate brachytherapy (LDR), and high dose rate brachytherapy (HDR) were converted to binary covariates as response variables.

Treatment delivery codes were determined based on the 2016 Edition of the American Society for Radiation Oncology (ASTRO) Radiation Oncology Coding Resource^7^ and are referenced in Table 1.

**TABLE 1:** HCPCS codes corresponding to delivery of radiation oncology procedures

HCPCS code	RT procedure
77401	3DCRT
77402	3DCRT
77407	3DCRT
77412	3DCRT
G6003	3DCRT
G6004	3DCRT
G6005	3DCRT
G6006	3DCRT
G6007	3DCRT
G6008	3DCRT
G6009	3DCRT
G6010	3DCRT
G6011	3DCRT
G6012	3DCRT
G6013	3DCRT
G6014	3DCRT
77385	IMRT
77386	IMRT
G6015	IMRT
G6016	IMRT
77371	SRS
77332	SRS
77373	SBRT
77778	LDR
77767	HDR
77768	HDR
77770	HDR
77771	HDR
77772	HDR

The total number of daily RT delivery codes submitted by each individual physician between January 1, 2016 and December 31, 2016 were summed. As the intent of this study was to examine variation in the delivery of RT procedures specifically, evaluation and management (E&M) codes were not included in this analysis.

To preserve patient privacy, CMS does not include any line item performed for 10 or fewer Medicare beneficiaries. Therefore, physicians submitting charges for 10 or fewer patients were excluded from analysis. Neither the POSPUF nor the PC database includes reimbursement information from other payers, demographic information about beneficiaries treated, or information about disease site treated.

Given that hospital‐affiliated radiation oncologists are not allowed to bill for technical services, (including procedures), the scope of this study is limited to non‐facility based (NFB) practice settings, which include freestanding outpatient clinics and federally qualifying health centers. Facility‐based (FB) billing patterns, such as those in hospital‐based practices including academic medical centers, were not studied.

Institutional Review Board approval was not required for analysis of this publicly available dataset that involved no human subjects.

### 2.2 Provider characteristics

Provider gender was reported directly in both the POSPUF database and the PC database. Where discrepancies existed, gender from the POSPUF database was used.

Practice rurality for each provider was classified as either metropolitan or non‐metropolitan. These definitions were based on the US Department of Agriculture classification using the Rural‐Urban Continuum Code (RUCC). Codes from 1 to 3 correspond to metropolitan areas whereas codes from 4 to 9 correspond to non‐metropolitan areas. To ascertain the RUCC, physician zip codes were mapped to a FIPS county code, which was then applied to the RUCC classification.

Practice region for each provider was drawn from the state codes in POSPUF and classified according to the four regions defined by the US Census Bureau^8^ : Northeast (reference region), Midwest, South, and West.

Physician credentials were divided into providers with an MD‐only degree and those with any combination of degrees other than MD‐only, which would include providers with MD‐PhDs, MD‐MPHs, MD‐MBAs, DO degrees, as well as foreign medical degrees.

Years since graduation, considered as a continuous variable, was defined as the number of years between the physician's graduation from medical school and the year 2016.

### 2.3 Statistical analysis

Descriptive statistics characterized gender, practice rurality, practice region, and credentials of radiation oncology providers.

Multivariable logistic regression analysis was used to determine provider characteristics that predict for the use of procedures in radiation oncology, considering the use of each RT delivery procedure to be an independent event. Covariates for multivariable regression analysis included provider gender (male vs. female), practice rurality (metro vs. non‐metro), practice region (Midwest, South, and West vs. Northeast), physician credentials (Degree other than MD alone vs. MD alone), and years since graduation as continuous variable. The overall significance of categorical variables in the multivariable logistic regression model was assessed by the likelihood ratio test (LRT).

Within‐subject (NPI) correlation was not addressed. For all statistical tests, the significance level was set at 0.05. All analyses were carried out in R 3.3.2.^9^

## 3 RESULTS

### 3.1 Identification of radiation oncologists

In total, 4,393 unique NPIs classified as radiation oncologists were identified in the POSPUF database. A total of 4,627 unique NPIs were classified as radiation oncologists in the PC database. Linking the two databases yielded 4,040 unique NPIs for analysis after 353 were eliminated because they failed to appear in both databases. A further 2,003 NPIs were eliminated because they lacked the pre‐specified HCPCS codes of interest. Finally, 235 NPIs were eliminated due to missing metropolitan status, leaving a total of 1,802 physicians available for analysis (Figure 1).

### 3.2 Demographics of included radiation oncologists

As shown in Table 2, of the 1,802 physicians, 1,383 (76.7%) were male and 419 (23.3%) were female. 119 (6.6%) practiced in rural areas while 1683 (93.4%) practiced in metropolitan areas. A total of 378 (21.0%) providers practiced in the Midwest, 290 (16.1%) in the Northeast, 743 (41.2%) in the South, and 391 (21.7%) in the West. Mean time from medical school graduation was 26.6 years (SD 11.1).

**TABLE 2:** Demographics of evaluated radiation oncologists

	Number (%)
Gender	
Male	1383 (76.7%)
Female	419 (23.3%)
Practice rurality	
Rural	119 (6.6%)
Metro	1683 (93.4%)
Practice location	
Midwest	378 (21.0%)
Northeast	290 (16.1%)
South	743 (41.2%)
West	391 (21.7%)
Credentials	
MD‐Only Degree	1656 (91.9%)
Combined or Non‐MD Degree	118 (6.5%)
NA	28 (1.6%)
	**Mean (SD)**
Years Since graduation	26.6 (11.1)

### 3.3 Predictors of RT procedure delivery

Table 3 demonstrates that on multivariable regression analysis, male gender predicted for increased LDR brachytherapy use (OR 8.19, 95% CI 2.58–26.05, *p* < 0.001), but not the use of other technologies. Metropolitan practice was the only predictor for greater HDR brachytherapy utilization (OR 12.95, 95% CI 1.81–92.60, *p* = 0.01).

**TABLE 3:** Multivariable analysis predicting RT procedure use

	Odds ratio	Lower CI	Upper CI	*p*‐value
**3DCRT**				
(Intercept)	0.67	0.47	0.97	0.03
Gender: Male vs. Female	0.94	0.81	1.09	0.38
Region: Midwest vs. Northeast	1.11	0.88	1.39	0.37
**Region: South vs. Northeast**	**1.33**	**1.09**	**1.62**	**<0.01**
**Region: West vs. Northeast**	**1.38**	**1.11**	**1.71**	**<0.01**
Years Since Graduation	1.00	1.00	1.01	0.45
Degree: Other than MD alone vs. MD alone	1.07	0.83	1.38	0.60
Rurality: Metro vs. Non‐metro	0.81	0.62	1.05	0.12
**IMRT**				
(Intercept)	0.31	0.20	0.46	<0.001
Gender: Male vs. Female	1.08	0.91	1.28	0.37
Region: Midwest vs. Northeast	0.99	0.77	1.27	0.93
Region: South vs. Northeast	1.03	0.83	1.28	0.77
Region: West vs. Northeast	1.03	0.81	1.30	0.83
Years Since Graduation	1.01	1.00	1.01	0.11
Degree: Other than MD alone vs. MD alone	1.19	0.90	1.57	0.21
Rurality: Metro vs. Non‐metro	0.96	0.72	1.28	0.78
**SRS**				
(Intercept)	0.62	0.41	0.95	0.03
Gender: Male vs. Female	0.89	0.74	1.06	0.19
**Region: Midwest vs. Northeast**	**0.71**	**0.55**	**0.91**	**<0.01**
**Region: South vs. Northeast**	**0.49**	**0.40**	**0.61**	**<0.001**
**Region: West vs. Northeast**	**0.43**	**0.34**	**0.55**	**<0.001**
Years Since Graduation	1.00	0.99	1.00	0.32
Degree: Other than MD alone vs. MD alone	0.74	0.53	1.04	0.08
Rurality: Metro vs. Non‐metro	0.88	0.64	1.21	0.43
**SBRT**				
(Intercept)	0.01	0.00	0.04	<0.001
Gender: Male vs. Female	1.07	0.75	1.54	0.71
**Region: Midwest vs Northeast**	**2.63**	**1.13**	**6.13**	**0.03**
**Region: South vs. Northeast**	**3.44**	**1.58**	**7.49**	**<0.01**
**Region: West vs. Northeast**	**4.87**	**2.21**	**10.72**	**<0.001**
**Years Since Graduation**	**0.98**	**0.96**	**0.99**	**<0.001**
Degree: Other than MD alone vs. MD alone	0.83	0.44	1.56	0.56
Rurality: Metro vs. Non‐metro	2.19	0.80	6.00	0.13
**LDR Brachytherapy**				
(Intercept)	<0.001	<0.001	<0.01	<0.001
**Gender: Male vs. Female**	**8.19**	**2.58**	**26.05**	**<0.001**
Region: Midwest vs. Northeast	0.98	0.45	2.17	0.97
Region: South vs. Northeast	1.33	0.69	2.58	0.40
Region: West vs. Northeast	0.68	0.31	1.52	0.35
**Years Since Graduation**	**1.02**	**1.00**	**1.04**	**0.02**
Degree: Other than MD alone vs. MD alone	0.72	0.26	2.01	0.53
Rurality: Metro vs. Non‐metro	2.41	0.75	7.73	0.14
**HDR Brachytherapy**				
(Intercept)	<0.01	<0.001	<0.03	<0.001
Gender: Male vs. Female	0.92	0.67	1.27	0.61
**Region: Midwest vs. Northeast**	**1.97**	**1.11**	**3.49**	**0.02**
Region: South vs. Northeast	1.63	0.96	2.76	0.07
**Region: West vs. Northeast**	**1.87**	**1.08**	**3.24**	**0.03**
Years Since Graduation	0.99	0.98	1.00	0.08
Degree: Other than MD alone vs. MD alone	1.11	0.65	1.90	0.70
**Rurality: Metro vs. Non‐metro**	**12.95**	**1.81**	**92.60**	**0.01**

Abbreviations: CI, Confidence interval.

In terms of geographic variation, as shown in Table S1, region was found to be predictive of 3DCRT, SRS and SBRT use (*p* < 0.01, *p* < 0.001, and *p* < 0.001, respectively). With Northeast as the reference region, 3DCRT was more likely to be delivered by providers in the South (OR 1.33, 95% CI 1.09–1.62, *p* < 0.01) and the West (OR 1.38, 95% CI 1.11–1.71, *p* < 0.01) compared to providers in the Northeast. At the same time, SRS was less likely to be delivered in the Midwest (OR 0.71, 95% CI 0.55–0.91, *p* < 0.01), South (OR 0.49, 95% CI 0.40–0.61, *p* < 0.001), and West (OR 0.43, 95% CI 0.34–0.55, *p* < 0.001) relative to the Northeast. SBRT, on the other hand, was more likely to be utilized in the Midwest (OR 2.63, 95% CI 1.13–6.13, *p* = 0.03), South (OR 3.44, 95% CI 1.58–7.49, *p* < 0.01), and West (OR 4.87, 95% CI 2.21–10.72, *p* < 0.001) compared to the Northeast. HDR brachytherapy use was also more likely in the Midwest (OR 1.97, 95% CI 1.11–3.49, *p* = 0.02) and West (OR 1.87, 95% CI 1.08–3.24, *p* = 0.03) compared to the Northeast.

While the degree held by the billing physician did not predict for procedure utilization, greater years since graduation was related to lower SBRT use (OR 0.98, 95% CI 0.96–0.99, *p* < 0.001) and higher LDR brachytherapy use (OR 1.02, 95% CI 1.00–1.04, *p* = 0.02).

## 4 DISCUSSION

This study identifies predictors of variation in the delivery of the most common radiation oncology procedures in the United States. We find that the radiation oncology provider gender, region of practice, rurality of practice, and years since graduation were all significantly associated with the delivery of specific RT procedures.

Radiation oncology has consistently demonstrated the cost‐effectiveness of its procedures across numerous disease sites.^10^, ^11^, ^12^ Yet, given variation in utilization, there may still be opportunities to ensure that costly RT procedures are being used judiciously and effectively in the cancer population. Effective care might thus be defined as care that is influenced by factors specific to the patient and their disease but not factors related to the experience or geography of their oncologists. Further study must elicit whether the variation reported herein represents effective care (evidence for the superiority of a given procedure is available and that procedure is selected for the patient), preference‐sensitive care (evidence for the superiority of one procedure over another is not available and the best choice depends on how patients value the risks and benefits of the treatment options), or supply‐sensitive care (irrespective of the evidence for or against a given procedure, that procedure is selected for the patient due to availability/supply of the procedure or expertise in performing that procedure), as defined by investigators at The Dartmouth Atlas.^1^ Outside of this work, the radiation oncology community continues to debate the relative appropriateness of certain procedures over others. While this study does not establish the degree to which the variation we report is warranted, the emergence of nearly 13‐fold differences in the odds of undergoing a given radiotherapeutic procedure from one region to the next certainly invites speculation as to whether patient characteristics are solely driving practice patterns.

With regard to physician gender as a predictor of RT procedure use, an earlier study highlighted significant gender differences in the use of radiotherapy treatment planning codes,^13^ whereas our analysis of treatment delivery codes did not reveal any significant gender differences other than with regard to use of LDR brachytherapy. A likely explanation for these disparate findings lies in the distinction between RT planning and delivery codes. While planning codes are submitted once per treatment course, delivery codes are submitted daily after each RT session, meaning that twenty courses of single‐fraction SRS will yield the same number of delivery codes as a single course of 20 fraction IMRT. It is thus possible that a RT planning analysis would better uncover differences in overall practice style, whereas a RT delivery analysis better illustrates cumulative reliance on certain radiation technologies. The latter may consequentially abrogate any gender distinctions in the utilization of specific procedures. We also note that unlike the previously published RT planning analysis,^13^ the present study excluded providers in facility‐based practices where technical billing is not allowed. Thus, when considering the larger national population of radiation oncologists, there may in fact exist gender differences in the delivery of RT procedures that our study was unable to capture. This gender‐based discrepancy between studies might also be explained by the fact that the treatment planning analysis examined just LDR, IMRT, and 3DCRT. The current analysis, which includes a greater number of RT techniques, may diminish our ability to discern significant differences among categories. Nevertheless, the strength of the association between male radiation oncologists and billing for LDR brachytherapy services is noteworthy, particularly since the billing of HDR brachytherapy was not similarly gendered. Since LDR brachytherapy is primarily used in the treatment of prostate cancer, it may confirm that genitourinary radiation oncologists in community practices are more likely to be male, and that males in radiation oncology may have training, mentorship, or practice incentives that result in disproportionate utilization of LDR brachytherapy when compared to females. The manner in which these findings interact with the gender pay gap in radiation oncology,^14^, ^15^ remains to be elucidated.

Rurality of practice was also found to be a significant predictor of HDR brachytherapy use, with increased use associated with providers practicing in metropolitan regions. Since rural physicians comprised just 6.6% of the total physicians in our analysis, a large difference would be required to achieve statistical significance, which adds further emphasis to the magnitude of this disparity. The findings of Hong et al confirm that high‐volume brachytherapy providers, most often located in urban centers, treat a disproportionately high number of patients,^16^ which raises important questions about the accessibility of this critical therapeutic modality.^17^, ^18^ Furthermore, in a setting where 30% of rural radiation oncologists plan to retire in the next 5 years (vs. 17% in urban and suburban settings),^19^ our data further underscore the need to support the availability of quality radiation oncology procedures in rural settings.

Others reporting on the “urban‐rural paradox” of cancer care similarly describe an uneven distribution of HDR brachytherapy procedures performed in the United States, with a shortfall in rural areas^20^, ^21^ influencing the delivery of guideline‐concordant care for many patients. Attempts to circumvent HDR brachytherapy with hypofractionated external beam treatments have yielded mixed results^22^, ^23^; thus, until mature data demonstrate alternatives to HDR brachytherapy that do not compromise outcomes, accessibility of this important treatment should be prioritized. While some procedures such as prostate SBRT have been shown to be more concentrated in urban areas,^24^ SBRT for other disease sites may be offsetting this trend, as we uncovered no rurality trends in the delivery of SBRT overall.

Transitioning to geographic variation in the delivery of RT procedures, despite the fact that our regions of interest are larger than the well‐established hospital referral regions,^25^ we still uncovered significant regional variation in practice. SBRT appeared much more likely to be performed outside of the Northeast, whereas SRS was more likely in the Northeast than other regions. We do not interpret these results as evidence that some regions are more likely to be early adopters of new technology than others, but rather that that there are regional preferences for employing one technology over another that may be self‐perpetuating.^26^ While variables such as socioeconomic status and disease burden could not be accounted for in this analysis,^27^ we are not aware of any data that support a near fivefold increase in these variables across broadly defined regions of the United States, which suggests that other factors are likely contributing to the commensurate fivefold variation in procedure use reported herein.

Grant et al^28^ reported significant variation in the use of IMRT and identified male provider gender, practice region in the southern United States, and academic practice as predictors of IMRT use. Other studies have reported greater intensity of IMRT use by self‐referring urologists compared to urologists that do not self‐refer.^29^ Our study, masked to referral patterns and limited only to freestanding practices where technical billing was allowed, revealed less variation in the use of IMRT compared to other technologies, suggesting that this procedure is perhaps the most uniformly employed among all regions, genders, and practice experience levels across the United States.

The fact that degree held by the provider did not predict for procedure use is not surprising, as residency training, rather than training prior to residency, is more likely to dictate practice style^30^ in the NFB setting.

Interestingly, greater years since graduation was related to decreased SBRT delivery, and increased LDR brachytherapy delivery. The former might be explained by a hesitation of late career physicians in community practice in carrying out higher dose‐per‐fraction procedures,^31^ or contrarily, overutilization by early career physicians based on more recent evidence of SBRT safety and efficacy across multiple disease sites^32^ as well as the oligometastatic setting.^33^, ^34^ Additional research is warranted to uncover whether missed opportunities exist for offering evidence‐based^32^, ^35^ hypofractionated regimens to patients, as well as elucidate the motivations of individual radiation oncologists^36^ at different stages of their career to select one treatment over another.

There are a number of limitations to claims‐based analyses such as ours. Our analysis is based exclusively on Medicare claims data, and the limitations of practice pattern studies in a payment environment that only represents a fraction of the US healthcare market are well‐established.^2^ The elimination of providers who performed a given procedure fewer than 10 times over the course of the study period may introduce bias to our analysis. This data is also restricted to enrollees who received coverage through fee‐for‐service Medicare plans and excludes Medicare Advantage plans as well as CMS authorized plans that are contracted out to private payers in the year 2016. Still, Medicare data can be useful and might even underestimate extremes of practice variation and spending, as some studies have even suggested the scale of variation is magnified outside of the Medicare system.^37^ While the elimination of facility‐based radiation oncologists from our study limits the generalizability of our findings, we still believe that in a small field such as radiation oncology, the practice patterns of over 1,800 physicians are still worth exploring because these data sharpen our understanding of billing practices in “real‐world” freestanding radiation oncology practices where technical billing is allowed. Prior work examining procedural planning codes in the larger radiation oncology population (including facility‐based providers) similarly uncovered significant variation in multiple practice settings,^13^ so it is clear that significant practice variation prevails irrespective of the practice setting studied. Moreover, this study is timely insofar as a shrinking proportion of practitioners in the NFB setting^19^ and a shift towards episodic billing^5^ may hinder future national practice pattern studies of this kind. And finally, while other practice pattern studies using the POSPUF have offered general overviews of the national billing landscape in radiation oncology,^38^ these less contemporaneous studies are also limited in that they did not stratify according to practice setting, where Medicare spending patterns tend to be drastically different.

In conclusion, other than LDR brachytherapy, provider gender does not relate to the daily delivery of RT procedures, though adopting newer RT techniques and technology appears less likely in providers farther out from training. Substantial geographic variation in the use of specific RT technologies was identified, and there remains a need to support the availability of quality radiation oncology in rural settings. The degree to which this variation reflects effective care, preference‐sensitive care, or supply‐sensitive care warrants further investigation. In the interim, training opportunities, for those who are interested, should be made available to enable the appropriate use of HDR brachytherapy and SBRT procedures.

## AUTHOR CONTRIBUTIONS

Each author has made substantial contributions to either the conception and design, acquisition of data, and/or analysis of data.

## Supporting information

Table S1

## Data Availability

All data used in this analysis are publically available from the Centers for Medicare and Medicaid Services.
