# Supplementary material for: National variation in the delivery of radiation oncology procedures in the non‐facility‐based setting
Source: Cancer Med. 2021 Jun 2;10(14):4734–42. doi: 10.1002/cam4.4028 (PMC8290244; doi:10.1002/cam4.4028)
Supplement: Supplementary file 1 — Table S1 [file CAM4-10-4734-s001.docx]

**Supplemental Table 1: Likelihood Ratio Test for Geographic Variation in Procedure Use**

|  | Resid. Df | Resid. Dev | Df | Deviance | Pvalue |
| --- | --- | --- | --- | --- | --- |
| 3D |  |  |  |  |  |
| model w/ region | 4212 | 5700.86 | NA | NA | NA |
| model w/o region | 4215 | 5714.14 | -3.00 | -13.28 | <0.01 |
| IMRT |  |  |  |  |  |
| model w/ region | 4212 | 4920.46 | NA | NA | NA |
| model w/o region | 4215 | 4920.70 | -3.00 | -0.25 | 0.97 |
| SRS |  |  |  |  |  |
| model w/ region | 4212 | 4199.09 | NA | NA | NA |
| model w/o region | 4215 | 4257.26 | -3.00 | -58.18 | <0.001 |
| SBRT |  |  |  |  |  |
| model w/ region | 4212 | 1427.12 | NA | NA | NA |
| model w/o region | 4215 | 1451.45 | -3.00 | -24.32 | <0.001 |
| LDR |  |  |  |  |  |
| model w/ region | 4212 | 842.61 | NA | NA | NA |
| model w/o region | 4215 | 848.20 | -3.00 | -5.59 | 0.13 |
| HDR |  |  |  |  |  |
| model w/ region | 4212 | 1668.77 | NA | NA | NA |
| model w/o region | 4215 | 1675.63 | -3.00 | -6.86 | 0.08 |
